# Spray-Dried Chitosan-Clay Nanocomposite Gels as Binders: Enhanced Compressibility and Micromeritic Properties of Lactose-Based Granules for Poorly Compressible Drug Tablets

**DOI:** 10.3390/pharmaceutics18101263

**Published:** 2026-10-06

**Authors:** Rapee Jarungsirawat, Peera Tabboon, Napaphak Jaipakdee, Duangkamon Sakloetsakun, Thaned Pongjanyakul

**Affiliations:** 1 Division of Pharmaceutical Technology, Faculty of Pharmaceutical Sciences, Khon Kaen University, Khon Kaen 40002, Thailand; rapee@kku.ac.th (R.J.); peerata@kku.ac.th (P.T.); nj.jaipakdee@gmail.com (N.J.); sduangk@kku.ac.th (D.S.); 2 Center for Research and Development of Herbal Health Products, Khon Kaen University, Khon Kaen 40002, Thailand

**Keywords:** chitosan, clay, nanocomposites, binder, lactose monohydrate, herbal crude tablets

## Abstract

**Background/Objectives:** Nanocomposites of chitosan (CH) and magnesium aluminum silicate (MAS) clay can be produced by spray drying. This study aimed to characterize and evaluate spray-dried chitosan (SDCH) and SDCH-MAS nanocomposites, which were prepared using CH with MWs of 400 kDa (4M) and 800 kDa (8M), as binders of lactose granules containing propranolol HCl (PPN) and crude powder from *Andrographis paniculata* (ADP) intended for tablet formulation. **Methods:** Lactose granules were produced using SDCH and SDCH-MAS composites via wet granulation, and the granule properties were compared with those obtained using polyvinylpyrrolidone (PVP K30). The lactose granules incorporating PPN and ADP crude powder were also prepared and compressed into tablets. The physical properties of both granules and tablets were evaluated. **Results:** SDCH-MAS composites formed intercalated or exfoliated nanocomposites, as proved by PXRD, and rapidly hydrated in water to form gels that functioned as binders. Lactose granules prepared with SDCH and SDCH-MAS composites exhibited fair to good flowability and demonstrated lower yield pressure and yield strength during plastic deformation than those prepared with PVP K30. Moreover, granules loaded with PPN offered acceptable flowability and could be compressed into tablets. Increasing MAS content reduced the disintegration time of tablets in 0.1 N HCl. The use of SD4MCH in tablets could retard PPN release, while the addition of MAS accelerated drug release. For ADP crude tablets with a 75:25 ADP-to-lactose ratio, acceptable hardness and friability were achieved when SD4MCH-MAS 1:0.6 composites and PVP K30 were used, and 75% of andrographolide dissolved within 30 min. **Conclusions:** SDCH-MAS nanocomposite gels are effective binders for lactose granules in wet-granulated tablets.

## 1. Introduction

Tablets are the most common dosage form for delivering active pharmaceutical ingredients (APIs), offering convenience, ease of use, and precise dose delivery with high accuracy [1]. However, the formulation of tablets presents significant technical challenges. APIs, herbal powders with high fiber content, and other components often lack the necessary properties for successful tableting, such as adequate flowability and compressibility [2,3,4,5]. To produce tablets with desirable physical and mechanical properties, pharmaceutical excipients such as diluents, disintegrants, lubricants, and binders are essential [6]. Among these, binders are particularly vital in tablet manufacturing, especially during the wet granulation process [1,7,8,9]. Wet granulation is a widely used technique in tablet production, especially for active ingredients with poor flow and compressibility, as it enhances their properties to facilitate tableting [10,11,12]. Effective binders that promote strong interparticle bonding can enable a reduction in the total amount of excipient content (binder and filler) required to achieve the desired tablet strength and physical properties [13]. The type and concentration of the binder have a significant impact on the compressibility and overall characteristics of the resulting granules and tablets [14,15].

Chitosan (CH) is a natural cationic biopolymer derived from chitin, consisting of D-glucosamine and N-acetyl-D-glucosamine units. CH is classified as a weak base, with a pK_a_ value for the D-glucosamine residues ranging between 6.2 and 7.0 [16]. As a result, CH is insoluble at neutral and alkaline pH levels. However, in acidic conditions, CH becomes soluble because the amino groups are protonated [17]. Due to its low toxicity, good biocompatibility, and biodegradability, CH has gained attention as a promising pharmaceutical excipient. It has been studied for various pharmaceutical applications, particularly as a tablet binder [18,19]. Previous research has demonstrated the effectiveness of CH as a binder in the wet granulation technique and compared its binding efficiency to that of other common cellulosic binders, such as hydroxypropyl methylcellulose and methylcellulose [18]. While CH exhibits limitations in water solubility and compressibility, especially during wet granulation, processing techniques like spray-dried chitosan (SDCH) in the presence of acetic acid can lead to particles with improved water solubility [20,21]. This improvement is attributed to the conversion of CH into its more soluble salt form, chitosan acetate [21].

CH has the potential to interact with negatively charged clays through electrostatic interactions and intermolecular hydrogen bonding, resulting in the formation of nanocomposites without the need for heat treatment in the CH-clay composite dispersions [22,23,24]. Montmorillonite, specifically magnesium aluminum silicate (MAS), is a pharmaceutical clay which is regarded as nontoxic at the level of use as an excipient. It has a silicate layer structure with silanol groups and has been reported to form a nanocomposite material with CH [25]. The creation of CH-MAS nanocomposites could enhance the thermal and mechanical properties of CH films [25,26]. Moreover, CH-MAS nanocomposites produced via the spray drying method can serve as matrix formers [27,28] and microparticle systems [29] for sustained drug release. Matrix tablets prepared with CH-MAS nanocomposites at ratios of 1:0.6 and 1:1 showed good hardness and sustained drug-release profiles in an acidic medium [27]. Regarding toxicity, CH-MAS nanocomposites at a 2:1 ratio showed low toxicity and good biocompatibility in the range of 5–500 mg/mL and effectively stimulated cell proliferation [30]. Therefore, it is worthwhile to explore the alternative use of SDCH-MAS nanocomposites as binders for preparing API granules using the wet granulation method. The binder properties of SDCH for preparing tablets have been reported previously [21]; thus, the use of SDCH-MAS nanocomposites may offer better characteristics for tablets due to their improved binder properties.

Lactose is the most commonly used filler or diluent in tablets due to its cost-effectiveness, availability, bland taste, low hygroscopicity, excellent physical and chemical stability, and water solubility [31]. It exists in different polymorphs (α-lactose monohydrate, anhydrous α-lactose, and anhydrous β-lactose), which possess distinct physical properties [32]. The most stable crystalline form of lactose is α-lactose monohydrate; however, it has relatively poor binding properties, primarily due to fragmentation under compression [33,34]. Its flowability is also poor, with a Carr’s index value of 34.67% [35]. Enhancing flowability and compressibility can be achieved through particle enlargement using binders [35]. Thus, it is interesting to use SDCH and SDCH-MAS nanocomposites for this purpose.

Incorporating drug powders into lactose granules was necessary to assess the properties of the granules and the resulting compressed tablets. Propranolol HCl (PPN), a water-soluble, non-selective beta-adrenergic antagonist used in hypertension management, exhibited poor flowability, as indicated by a Carr’s index of 32.95% and a repose angle of 51.88 degrees. Compressed tablets containing pure PPN displayed broken edges after compression [36]. The herbal crude powder of *Andrographis paniculata* (ADP), derived from dried aerial parts and containing several diterpenoid lactones and flavonoids, demonstrated antiviral activity against severe acute respiratory syndrome coronavirus 2 [37]. ADP crude powder also exhibited low bulk density and poor flowability, with a Carr’s index of 40.02% [38]. These findings indicate that both PPN and ADP crude powders are poorly compressible materials. Consequently, they were selected to evaluate the performance of SDCH and SDCH-MAS nanocomposites as binding agents, with lactose serving as a diluent.

This study represents the first investigation into the use of SDCH-MAS nanocomposites as binders in the wet granulation method. The primary objectives were to evaluate the effects of SDCH and SDCH-MAS composites on the micromeritic properties and compressibility of lactose granules. SDCH and SDCH-MAS composites, made with different MWs of CH, were prepared by spray drying. The swelling and rheological properties of these composites in water were examined. Lactose granules were produced utilizing SDCH and SDCH-MAS composites as binders through the wet granulation method. For comparison, PVP K30, a commonly used pharmaceutical binder, was employed. Lactose granules incorporating PPN and ADP crude powder were also prepared. The micromeritic properties, compressibility, and flowability of the granules were evaluated. Additionally, the PPN and ADP crude granules were compressed into tablets, and their properties, including weight, thickness, disintegration time, and drug dissolution behavior, were characterized.

## 2. Materials and Methods

### 2.1. Materials

CH with MW 400 kDa and 800 kDa (4MCH and 8MCH), which were 85% degree of deacetylation) were sourced by Bio21 Co., Ltd. (Chonburi, Thailand). MAS in granular form and lactose in monohydrate form were manufactured by R.T. Vanderbilt Company, Inc. (Norwalk, CT, USA) and Thai Meochems Co., Ltd. (Bangkok, Thailand). ADP crude powder was purchased from Vejpong Pharmacy (Hock An Tang) Co., Ltd. (Bangkok, Thailand). The andrographolide content, extracted using methanol and analyzed by high performance liquid chromatography (HPLC), was 1.83 ± 0.18% *w*/*w*, n = 3. PPN and PVP K30 were obtained from Changzhou Yabang Pharmaceutical Co., Ltd. (Changzhou, Jiangsu, China) and Union Sciences Trading Co., Ltd. (Khon Kaen, Thailand). Magnesium stearate was purchased from Sigma-Aldrich Co. (St. Louis, MO, USA). All other materials were used as received.

### 2.2. Preparation of SDCH-MAS Nanocomposites

A 1.5% *w*/*v* CH dispersion in 1% *w*/*v* acetic acid was prepared by stirring at room temperature (26–28 °C) for 12 h. The 3% *w*/*v* MAS suspension was produced using hot water (temperature > 85 °C) and then cooled down to room temperature. Next, the 3% *w*/*v* MAS suspension was diluted with 10 mM acetate buffer at pH 4 to achieve a 1.5% *w*/*v* MAS dispersion. After that, 1.5% *w*/*v* CH and 1.5% *w*/*v* MAS dispersions were mixed in various volumes to obtain CH-MAS ratios of 1:0, 1:0.6, and 1:1, with the solid content of the final mixture constant at 1.5% *w*/*v*. Glacial acetic acid was used to adjust the final pH of the mixtures to 4. The mixtures were then left at room temperature for 24 h before spray drying. A spray dryer (Mobile MINOR™ GEA Niro A/S, Soeborg, Denmark) was used, with the spray-dried condition set at an inlet temperature of approximately 150 °C and an outlet temperature of 83 ± 3 °C. The air pressure for feeding was 80 kPa, and the feeding rate of the mixtures was 1000 mL/h. Exhaust air was taken out from the chamber and generally led to a cyclone followed by a bag filter. The SDCH and SDCH-MAS composites were collected from a product separator and stored in a desiccator containing silica gel beads before testing.

### 2.3. Characterization of SDCH and SDCH-MAS Nanocomposites

#### 2.3.1. Rheological Behavior and Viscosity

SDCH and SDCH-MAS composites (1 g each) were dispersed in 100 mL of purified water to create a 1% *w*/*v* gel. The rheological properties and viscosity of these gels were evaluated using a Brookfield viscometer (Model LTV, Brookfield Engineering Laboratory Inc., Middleboro, MA, USA) equipped with a small sample adapter. A 10 mL sample was used, and the temperature was maintained at 30 °C. Spindle No. 31 was employed to measure the shear stress. A rheogram, which illustrates the relationship between shear rate and shear stress, was generated by recording various spindle revolutions. To characterize the flow type of the samples, the following exponential formula was utilized [39,40]:

F^N^ = ηG (1)

Log G = N Log F − Log η(2)

where F, G, N, and η are shear stress, shear rate, exponential constant and viscosity coefficient, respectively. In addition, the apparent viscosity at 20.4 s^−1^ shear rate was employed for comparisons.

#### 2.3.2. Scanning Electron Microscopy (SEM)

The particle size and morphology of the SDCH and SDCH-MAS composites were analyzed using scanning electron microscopy (SEM). The samples were attached to a metallic stub with carbon adhesive tape. Subsequently, they were coated with gold before being examined using a Hitachi S-3000N scanning electron microscope (Tokyo, Japan).

#### 2.3.3. Powder X-Ray Diffractometry (PXRD)

The PXRD patterns of the MAS, CH, SDCH, and SDCH-MAS composites were investigated using a powder X-ray diffractometer (Malvern Panalytical Empyrean, Malvern, Worcestershire, UK). The Cu Kα radiation was generated at 40 kV and 40 mA. The X-ray scanning was done over the range of 5°–25° 2θ with an angle step of 0.02° 2θ/s. The interlayer distance of the MAS silicate layers was calculated using Bragg’s equation: nλ = 2dsinθ, where n is 1 (the first order reflection was used), λ is the X-ray wavelength (0.154 nm), θ is the angle of the basal spacing peak of MAS, and d is the interlayer distance of the MAS silicate layer [25,26].

### 2.4. Preparation of Lactose Granules Using SDCH, SDCH-MAS and PVP K30

Lactose granules were prepared using SDCH, SDCH-MAS composites, and PVP K30 as a binder. The formulations were composed of 98% *w*/*w* lactose monohydrate and 2% *w*/*w* binder. The batch size for this preparation was 100 g. The lactose monohydrate (98 g) was mixed with 2 g of binder using a mortar and pestle for 5 min. Purified water (20–25 mL) was then continuously sprayed into the powder mixtures, which were mixed for 15 min and kneaded with a pestle until a damp mass formed. The damp mass was sieved through sieve no. 12 (1.7-mm mesh). The resulting wet granules were dried in a hot air oven at 55 °C for 2–3 h until the weight of the dried granules was constant and the moisture content was 4–6% *w*/*w*. Next, the particle size of the dried granules was reduced by a secondary sieving through sieve no. 18 (1-mm mesh). Finally, the granules were stored in a desiccator containing silica gel beads before characterization.

### 2.5. Evaluation of Lactose Granules

#### 2.5.1. Density and Flowability

The flow properties of the lactose granules were assessed based on Carr’s Index and angle of repose. To determine Carr’s Index, both the bulk and tapped densities were measured. A weight of 10 g of granules was gently added to a 50 mL measuring cylinder, and the bulk volume was recorded to calculate bulk density (D_B_). The cylinder with the granules was then tapped from a height of 1.5 inches until the volume of the granules remained constant. The final volume was noted to compute the tapped density (D_T_). The Carr’s index value was calculated using the formula [41]: Carr’s index (%) = 100 × (D_T_ − D_B_)/D_T_. For the angle of repose, the granules were gradually poured through a glass funnel onto a flat surface at a constant height of 4 cm. The orifice inner diameter of the glass funnel was 10.2 mm. The height (h) and radius (r) of the resulting pile were measured, and the angle of repose was calculated using the formula [42]: repose angle (°) = arctan (h/r).

The true density of the lactose granules was determined using a liquid displacement method with a 5-mL pycnometer, utilizing acetone as the liquid to ensure that the granules’ ingredients would not dissolve in it. A weighed pycnometer was filled with 1 g of the granules and weighed accurately. Acetone was then added to fill the pycnometer containing the granules to the maximum volume, and the total weight of the filled pycnometer was recorded. The weight of the acetone in the pycnometer without the sample was also determined. The true density was calculated using the following equation [43]: true density = (W_A_ × W_G_)/(5 × (W_A_ − W_GA_ + W_G_)), where W_A_, W_G_, and W_GA_ represent the weights of acetone, granules, and granule-acetone mixture in the pycnometer, respectively.

#### 2.5.2. Compressibility

The lactose granules (300 mg) without lubricant were compressed into tablets at various compression pressures, using a hydraulic press (Retsch™ PP 25 Pellet Press, Haan, Germany) with applied pressures ranging from 31.2 to 124.9 MPa. Three tablets were used for each compression pressure. The out-die thickness of each tablet was measured immediately after ejection using a digital Vernier caliper with an accuracy of ±0.01 mm. The tablet density and volume under compression pressure were calculated. Furthermore, the tensile strength of the tablets obtained was also calculated using the equation: tensile strength (σ_t_) = 2F/πDT [44,45], where F is the break force of flat-faced tablets subject to diametrical compression (referring to a tablet’s hardness), D is a tablet’s diameter, T is a tablet’s thickness and π is a constant of 3.143.

The Heckel analysis, which describes the compression behavior of a material in terms of its relative density under applied compression pressure, was used in this study. The foundation of the Heckel plot for powder compression is that the densification of the bulk powder under pressure follows first-order kinetics. This relationship is expressed by the following equation [46]:

ln (1/(1 − D)) = KP + A(3)

where D represents the relative density of the tablet (the ratio of tablet density to the true density of the granule) under compression pressure (P). K is the slope of the linear portion of the Heckel plot, and the reciprocal of K provides the mean yield pressure (P_y_). The relationship between ln (1/(1 − D)) and P was plotted, and the slope and intercept constants were determined [46,47,48]. Moreover, the constant A accounts for the densification of the granule due to initial particle rearrangement (D_a_), which can be calculated using an equation:

D_a_ = 1 − e^−A^ (4)

The Kawakita analysis is applied to characterize particle deformation under pressure by plotting the relationship between compression pressure and powder volume change. The Kawakita equation was presented as follows [47,48]:

P/C = P/a + 1/ab(5)

C = (V_0_ − V)/V_0_ (6)

where P is the compression pressure, and C describes the volume decrease after applying compression pressure. V_0_ is the initial volume of powders filled into the die, which could be computed using the bulk density. V is the tablet volume after compression, which was determined from tablet thickness. The a value, indicative of the initial porosity, decreases with compression pressure. The b value is proposed to be inversely related to the yield strength of particles (P_k_). Data for P/C and P were plotted, and the slope (1/a) and intercept (1/ab) constants were obtained.

#### 2.5.3. Mechanical Strength of Lactose Granules

To evaluate the mechanical strength of the granules, large-particle-size lactose granules were prepared. The wet mass of lactose and binder was passed through a 1.7 mm screen and then dried in an oven at 55 °C for 2–3 h. Twenty granules were randomly selected for this measurement. An individual granule was pressed, and the maximum force was recorded at 50% probe displacement using a TA.XT Plus texture analyzer (Stable Micro Systems, Godalming, UK). The instrument was equipped with a 50-kg load cell and a 6-mm diameter cylindrical probe. A test specimen was positioned on the platform under ambient conditions. The probe was carefully placed to engage with the test specimen and subsequently descended at a steady rate of 1.0 mm/s. After reducing the distance to 50% of its initial height, the probe was retracted. The relationship between force and percentage displacement was plotted, and the maximum force at 50% displacement was reported, reflecting the mechanical strength of the granules [49].

### 2.6. Preparation of PPN Tablets via Wet Granulation Method

PPN tablets were prepared by wet granulation. Each tablet contained 80 mg of PPN and had a total weight of 300 mg. Lactose served as a diluent, while PVP K30, SDCH, or SDCH-MAS composites were used as binders at a concentration of 2% *w*/*w*. The formulations consisted of 26.7% PPN, 71.3% lactose monohydrate, and 2% binder by weight. The batch size for the preparation was 100 g. The preparation process began by mixing lactose and PPN for 5 min using a mortar and pestle. Afterward, the binder powder was added, and the mixture was blended for an additional 5 min. The mixture was wetted by spraying 35–40 mL of purified water, mixed and kneaded for 15 min to obtain a damp mass. The damp mass was passed through a No. 12 sieve (1.7-mm mesh) to produce wet granules, which were subsequently dried in a hot air oven at 55 °C for 2–3 h until a constant weight of the dried granules was obtained, and the moisture content of the granules was in the range of 4–6% *w*/*w*. To reduce the particle size of the dried granules, they were sieved using a No. 18 sieve (1.0-mm mesh). Magnesium stearate was added to the granules at a concentration of 1% *w*/*w* and mixed for 1 min in a roto-mixer. Finally, the PPN granule mixture was compressed into tablets using a hydraulic press (Retsch™ PP 25 Pellet Press, Haan, Germany) at a compression pressure of 125 MPa, with flat-faced punches and a 10 mm-diameter die.

### 2.7. Characteristics of PPN Granules and Tablets

#### 2.7.1. Micromeritic Properties of PPN Granules

The bulk and tapped densities, as well as particle flowability, were characterized using the methods outlined in Section 2.5.1.

#### 2.7.2. Thickness and Hardness of PPN Tablets

The hardness of the PPN tablet was evaluated using a hardness tester (Model 40–2100 VK200, VanKel^®^, Cary, NC, USA). The thickness of the tablets was measured with a digital caliper (Model DCS150, Kennedy^®^, Leicester, UK). Five tablets of each formulation were individually measured for thickness and hardness using the instruments mentioned above.

#### 2.7.3. PPN Content in Tablets

To determine the PPN content in the tablets, each tablet was dissolved in 100 mL of a 2 N HCl solution for 1 h. Then, the supernatant was collected and filtered through a 0.45-µm prefilter. PPN concentrations were analyzed using a UV–Visible spectrophotometer (Model UV1201, Shimadzu, Kyoto, Japan) at a wavelength of 289 nm. The relationship between absorbance values and PPN concentrations in the range of 5–50 μm/mL presented a linear relationship with R^2^ > 0.999.

#### 2.7.4. Disintegration and Release of PPN Tablets

The disintegration time was evaluated using a basket rack assembly disintegration test apparatus (Model ZT-324, Erweka America Inc., Edison, NJ, USA) at 37.0 ± 0.5 °C, using 0.1 N HCl as the medium. Each tablet was placed with an additional dish within the basket, and the disintegration time was recorded when the tablet was entirely removed from the basket sieve.

The drug release profile of the PPN tablets was assessed using a USP apparatus I (basket method) with a Vankel dissolution tester model VK-7000 (Cary, NC, USA). The basket rotation speed was maintained at 50.0 ± 1.0 revolutions/min, and the temperature was held at 37.0 ± 0.5 °C. The dissolution medium consisted of 750 mL of 0.1 N HCl. Samples were collected at each time interval by filtering using a cannula filter (pore size 10 μm) and replaced with equivalent volumes of fresh medium. The PPN content was analyzed using a UV–vis spectrophotometer (Shimadzu UV1201, Kyoto, Japan) at a wavelength of 289 nm. The absorbance values were plotted with PPN concentrations of 5–50 μm/ml and the linearity of this relationship was obtained with R^2^ > 0.999. The T_50%_ and T_75%_ values, which indicate the times required to achieve 50% and 75% drug release of the drug content, were calculated [49]. Three tablets of each formulation were individually determined for disintegration time and drug release.

The drug released from the tablets containing a hydrophilic polymer that showed a sustained-release profile was analyzed the drug release mechanism by using a power law [50,51,52], as presented in the equation:

M_t_/M_∞_ =kt^n^ (7)

where M_t_/M_∞_ is the fractional release, t is time, k is a release constant involving structural and geometric characteristics of the system, and n is the release exponent, indicating the mechanism of release. The n value of 0.5 denotes a diffusion-controlled drug release (Fickian diffusion), whereas a value of unity indicates a polymer swelling-controlled release mechanism. The n value within the range of 0.5–1 corresponded to anomalous transport, a combination of drug diffusion and polymer swelling mechanisms. The n and k values for each tablet were obtained by fitting a linear equation to the logarithm of the fractional release (*y*-axis) and the logarithm of time (*x*-axis) when the fractional release was less than 0.6. The slope of the equation is the release exponent n, while log k is the intercept [52].

### 2.8. Preparation of ADP Crude Granules and Tablets

Herbal tablets containing ADP crude powder were prepared by wet granulation, following the method described in Section 2.6. To determine the minimum amount of lactose (diluent) required for acceptable tablet formation, formulations were prepared with different ADP:lactose ratios of 100:0, 75:25, and 50:50 (by weight) with a binder content of 2% *w*/*w*. Table 1 lists the components and purified water used for granule preparation. The amount of purified water depended on the amount of ADP crude powder, which had a high water-holding capacity. The batch size for the granule preparation was 100 g. SD4MCH was used as the initial binder to evaluate its functionality. For tablet preparation, the granules obtained were mixed with 1% *w*/*w* magnesium stearate. The mixtures were mixed using a roto-mixer for 5 min. The total tablet weight was fixed at 500 mg. Tablets were compressed using a single-punch tableting machine (Model CMT-01, Charatchai Machinery Ltd., Part., Bangkok, Thailand) equipped with flat-faced punches and a 13-mm diameter die. To ensure consistent compression conditions across all formulations, the tablet thickness was tightly controlled at 3.2–3.4 mm. This parameter allowed for consistent compression and punch travel during tableting.

Based on preliminary evaluations, the ADP:lactose 75:25 formulation produced tablets with acceptable properties and was selected as the optimal ratio for further binder comparisons. Using this ratio, additional granules and tablets were prepared to evaluate the binding ability of SD4MCH-MAS composites and PVP K30, following the same granulation and compression procedures described above.

### 2.9. Characteristics of ADP Crude Granules and Tablets

#### 2.9.1. Micromeritic Properties and Moisture Content of ADP Granules

The bulk and tapped densities, as well as particle flowability, were characterized using the methods mentioned in Section 2.5.1. The moisture content of the granules was determined using a moisture analyzer (Aczet MB50 Moisture Analyzer, Mumbai, India). Approximately 2 g of the granules were placed in the sample pan and heated to 90 °C until constant weight was achieved. The percentage of moisture was automatically calculated based on the weight loss during the drying process.

#### 2.9.2. Evaluation of ADP Crude Tablets

Twenty tablets were weighed individually using a precision analytical balance. The mean, standard deviation, and relative standard deviation (%RSD) were calculated. The hardness and thickness of the tablet were measured using the methods described in Section 2.7.2.

For tablet friability, a friability tester (Model 45-2200, VanKel^®^, Cary, NC, USA) was used. Thirteen tablets weighing more than 6.5 g were placed in the drum and subjected to 100 revolutions over a 4-min period. After removing surface dust, the tablets were reweighed. The percentage friability was calculated using the formula: friability (%) = ((initial weight–final weight)/initial weight) × 100. A friability value of less than 1.0% was considered acceptable for uncoated tablets.

The disintegration time of the ADP crude tablets was measured using the same method described in Section 2.7.4. Dissolution testing was carried out using the USP Apparatus II (paddle method) (Vankel dissolution tester model VK-7000, Cary, NC, USA). The medium used was 500 mL of 0.1 N HCl at 37.0 ± 0.5 °C. The rotation speed of the paddle was 100.0 ± 1.0 revolutions/min. Samples were collected at predetermined time intervals by filtering using a 10 μm-pore size cannula filter and replaced with equivalent volumes of fresh medium. The samples were analyzed using HPLC for andrographolide in the ADP crude tablets. Three tablets were tested individually for ADP dissolution.

### 2.10. HPLC Analysis

The quantification of andrographolide in the ADP crude powder and tablets was performed using an HPLC assay following the previous report [53]. A C18 reversed phase column (250 × 4.6 mm, 5 µm, HiQ silTM HPLC Columns, KYA Technologies Corporation, Tokyo, Japan) was employed in conjunction with a model 1220 series HPLC system (Agilent^®^, Waldbronn, Germany). The chromatography was conducted using isocratic elution with a mixture of acetonitrile and 0.1% (*v*/*v*) phosphoric acid in water (40:60) at a flow rate of 1.0 mL/min. The working column temperature was established at 30 °C, the injection volume at 20 µL, and the UV detection wavelength at 223 nm. The retention time of andrographolide was approximately 4.7 min. The chromatographic technique exhibited robust linearity (R^2^ > 0.999) over the assessed concentration range (1–100 μg/mL).

### 2.11. Statistical Analysis

A student *t*-test and one-way analysis of variance with Tukey’s multiple comparison post hoc test (SPSS software for Microsoft Windows, version 28.0, IBM Corp., Armonk, NY, USA) were performed to determine the significant difference of maximum force at 50% displacement of lactose granules, and tablet properties such as hardness, disintegration time, and drug dissolution parameters. The significance level was set at 95% (*p* < 0.05).

## 3. Results and Discussion

### 3.1. Characteristics of SDCH-MAS Composites

**Figure 1:**
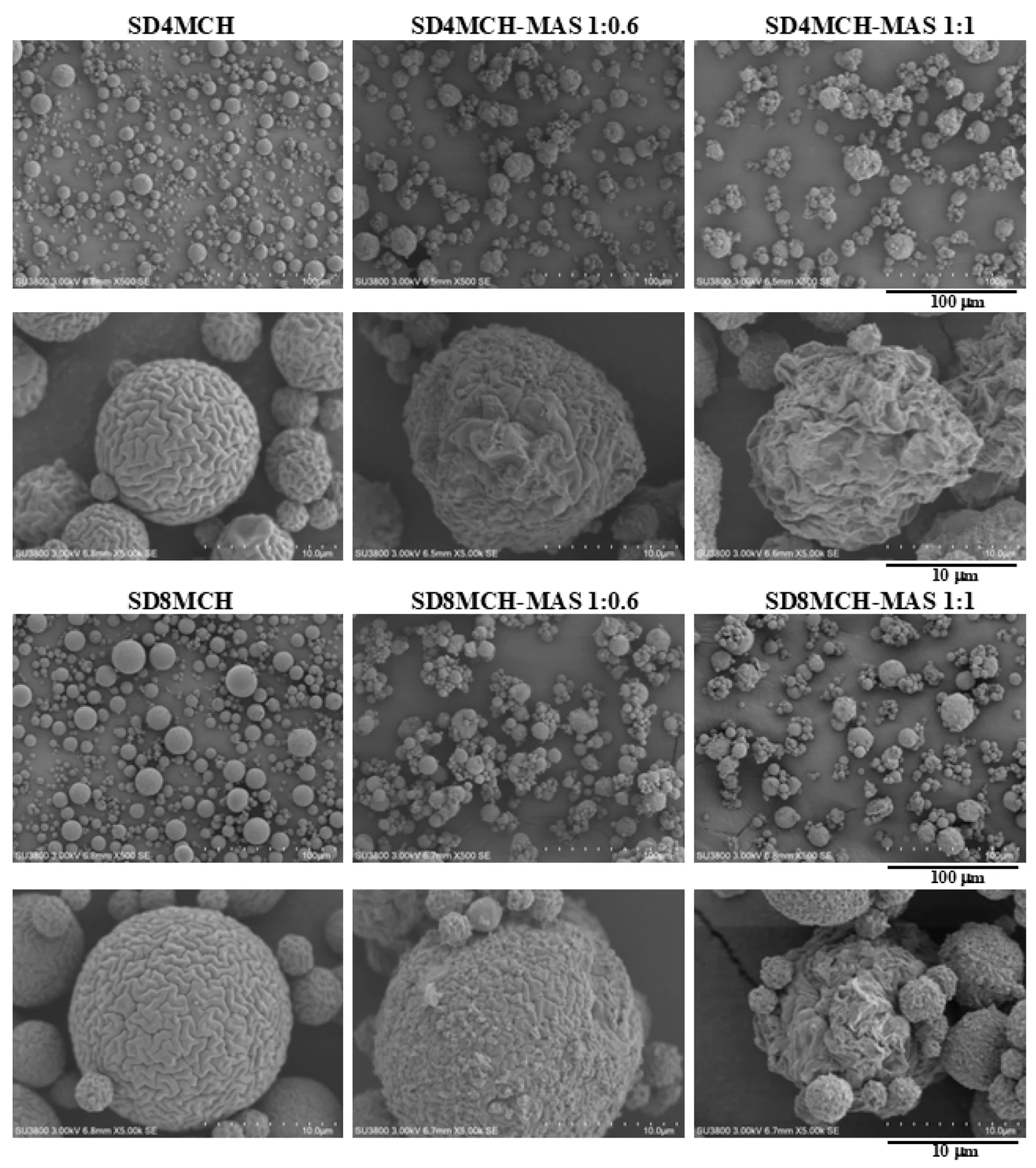
Particle morphology of SDCH and SDCH-MAS composites at the ratios of 1:0.6 and 1:1 using CH molecular weights of 400 kDa (4M) and 800 kDa (8M).

Particle and surface morphology of SDCH and SDCH-MAS composites prepared using spray drying are shown in Figure 1. Their particle sizes were in the range of 5–25 microns when observed from the SEM pictures. The high variation in particle size was observed because a pressure nozzle used in a spray dryer can produce a wide range of droplet sizes during spraying [54]. From the SEM images, the SD4MCH particles were considerably smaller than the SD8MCH particles. Both SD4MCH and SD8MCH were spherical particles with wrinkled surfaces. These features are caused by rapid solvent evaporation, resulting in particle collapse [55]. Adding MAS produced irregular particles in both MWs of CH used, and particle agglomeration increased with higher MAS content. Additionally, varying the MWs of CH did not impact the shape of the SDCH-MAS particles. The irregularity in the shape of the particle surfaces was attributed to a decrease in chitosan ratios while keeping the solid content in the feeding dispersions constant. Moreover, the complexation between CH and MAS, resulting from molecular interactions, may affect the dispersibility of CH in the composite particles.

**Figure 2:**
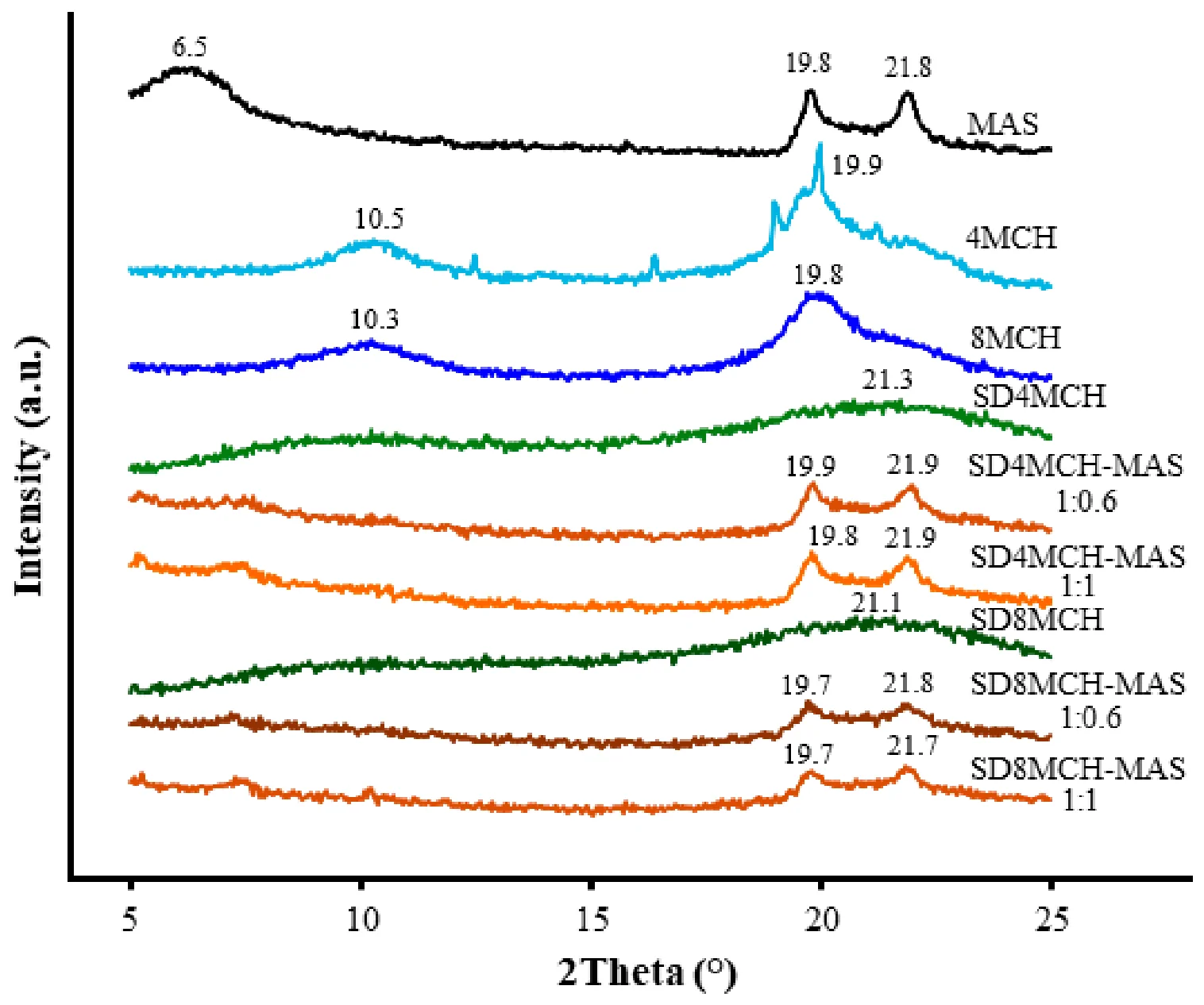
Powder X-ray diffractometry (PXRD) patterns of MAS, CH, SDCH, and SDCH-MAS composites at the ratios of 1:0.6 and 1:1 using CH molecular weights of 400 kDa (4M) and 800 kDa (8M).

To confirm the formation of a nanocomposite of CH and MAS, PXRD was used, and the patterns are shown in Figure 2. The MAS PXRD pattern showed clear peaks at 6.5, 19.8, and 21.8° 2θ. The basal spacing peak of MAS was located at 6.5° 2θ, indicating that the interlayer distance of MAS was 1.36 nm, which was similar to the previous report [25]. The 4MCH and 8MCH showed broad peaks around 10.4 and 19.8° 2θ, whereas the single broad peak at 21.2° 2θ was found for SD4MCH and SD8MCH. These results suggested that the spray-drying process decreased the crystallinity of CH. The PXRD patterns of the SDCH-MAS composites at different ratios displayed MAS peaks at approximately 19.8 and 21.8° 2θ, and the basal spacing peak of MAS disappeared. These outcomes indicated that the nanocomposite between CH and MAS was exfoliated, with complete separation of the MAS silicate layers in the CH matrix [27,56]. However, the basal spacing peak of the MAS silicate layers at 2θ below 5° may have arisen because the PXRD detection in this study was limited to 2θ = 5°. The interlayer distance of the MAS silicate layers was possibly greater than 1.36 nm, indicating an intercalated type of nanocomposite [56]. Thus, the SD4MCH-MAS and SD8MCH-MAS composites at 1:0.6 and 1:1 ratios were intercalated or exfoliated nanocomposites.

**Figure 3:**
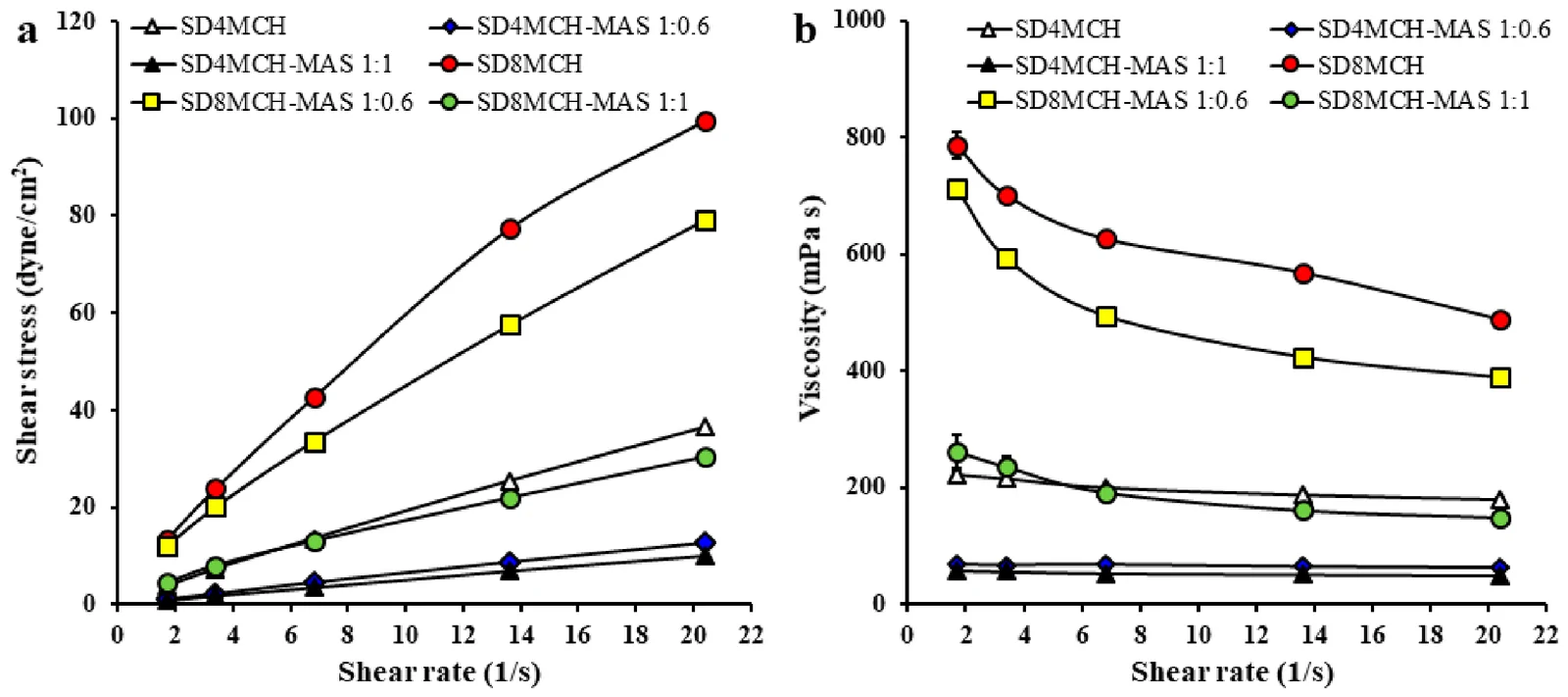
Rheograms (**a**) and relationship between shear rate and viscosity (**b**) of SDCH and SDCH-MAS gels. Each point represents the mean ± standard deviation (*n* = 3).

The SDCH and SDCH-MAS composites hydrated and swelled rapidly in water to form a viscous gel because the SDCH obtained was an acetate salt, as revealed by FTIR spectroscopy in the previous study [27]. The pH of SDCH and SDCH-MAS gels was in the range of 5.62–5.95, as listed in Table 1. These pHs were lower than the pK_a_ of CH (6.2–7.0 [16]), thus its molecule in the gels was in the protonated form, leading to hydration and disentanglement of a long side chain in water. Therefore, viscous gels were obtained. The flow behavior of the gel obtained was characterized in this study. The rheogram, which was a relationship between shear rate and shear stress, of the gels is presented in Figure 3a. The gel’s rheogram presented only an up curve because a down curve was the same as the up curve, suggesting the gel had no thixotropic properties. The N value and viscosity coefficient calculated from Equations (1) and (2) are listed in Table 2. The SD4MCH and SD4MCH-MAS gels exhibited an N value between 1.0 and 1.1, and the gel’s viscosity was quite constant when varying shear rate (Figure 3b), indicating Newtonian flow [39]. Moreover, the viscosity coefficient decreased with the addition of MAS. An increase in MW of CH caused a higher N value and viscosity coefficient of the SD8MCH and SD8MCH-MAS gels (Table 2). Furthermore, the viscosity of the gels decreased with increasing shear rate (Figure 3b). These results indicated a pseudoplastic flow behavior and higher viscosity at higher MW of CH [39]. However, the SD8MCH and SD8MCH-MAS 1:0.6 gels showed similar N values and viscosity coefficients. The viscosity coefficients of the gels could not be used for comparison because they depended on the N value. Thus, the apparent viscosity at a shear rate of 20.4 1/s was employed, as shown in Table 1. It was observed that higher MW CH resulted in greater gel viscosity, consistent with a previous report [57]. Furthermore, the addition of MAS was found to reduce the viscosity of the gels at both CH MWs. This finding suggests that CH and MAS could interact at the molecular level through electrostatic forces to form complexes. As a result, this interaction reduces the hydration and swelling properties of CH molecules. For this reason, the viscosity of the SDCH-MAS gels decreased as the MAS ratio increased.

### 3.2. Characteristics of Lactose Granules

SDCH and SDCH-MAS can be used as binders for preparing lactose granules because they hydrate and swell when exposed to water during wet mass production, as mentioned in Section 2.4. So, the granules could be successfully prepared in this study.

**Figure 4:**
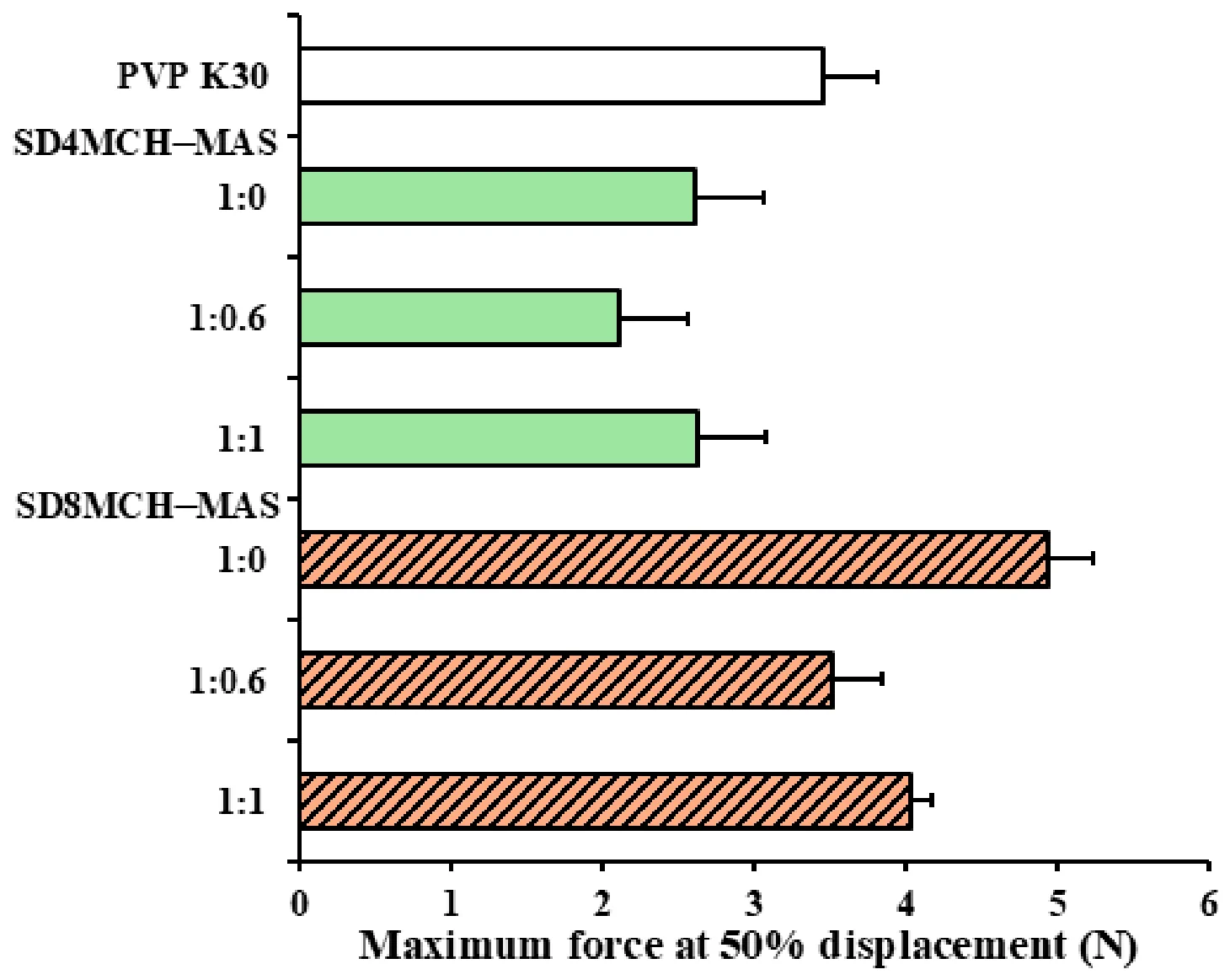
Maximum force at 50% displacement, mechanical strength, of lactose granules prepared using various binders. Each value represents the mean ± standard deviation (*n* = 20).

The maximum force at 50% displacement of the measured probe of the texture analyzer, which represented a mechanical strength of the lactose granules, is shown in Figure 4. The maximum force value of the granules using PVP K30 was 3.45 ± 0.36 N, *n* = 20, which was a significantly higher (*p* < 0.05) than those using SD4MCH (2.61 ± 0.44 N). The granules using SD8MCH (4.94 ± 0.29 N) showed significantly higher particle strength (*p* < 0.05) than those using PVP K30 and SD4MCH. This result suggested that the MWs of CH used influenced the particle strength of the granules. The higher MW polymer could be entangled to create a physical network, resulting in high adhesive strength for binding when compared to the lower MW polymer, as observed in the case of using PVP as a binder [58]. Similarly, increasing the length of the side chain of CH provided higher viscosity and a stronger binding force, inducing more robust interparticulate interactions between lactose particles to form granules. The reduction of CH by adding MAS resulted in a decrease in the strength of the granules, as observed with SD4MCH-MAS and SD8MCH-MAS at a 1:0.6 ratio. However, increasing the MAS amount at a 1:1 ratio tended to enhance the particle strength of the granules, suggesting that MAS and CH-MAS nanocomposites could increase the adhesion of the lactose particles, thereby creating a stronger granule.

**Figure 5:**
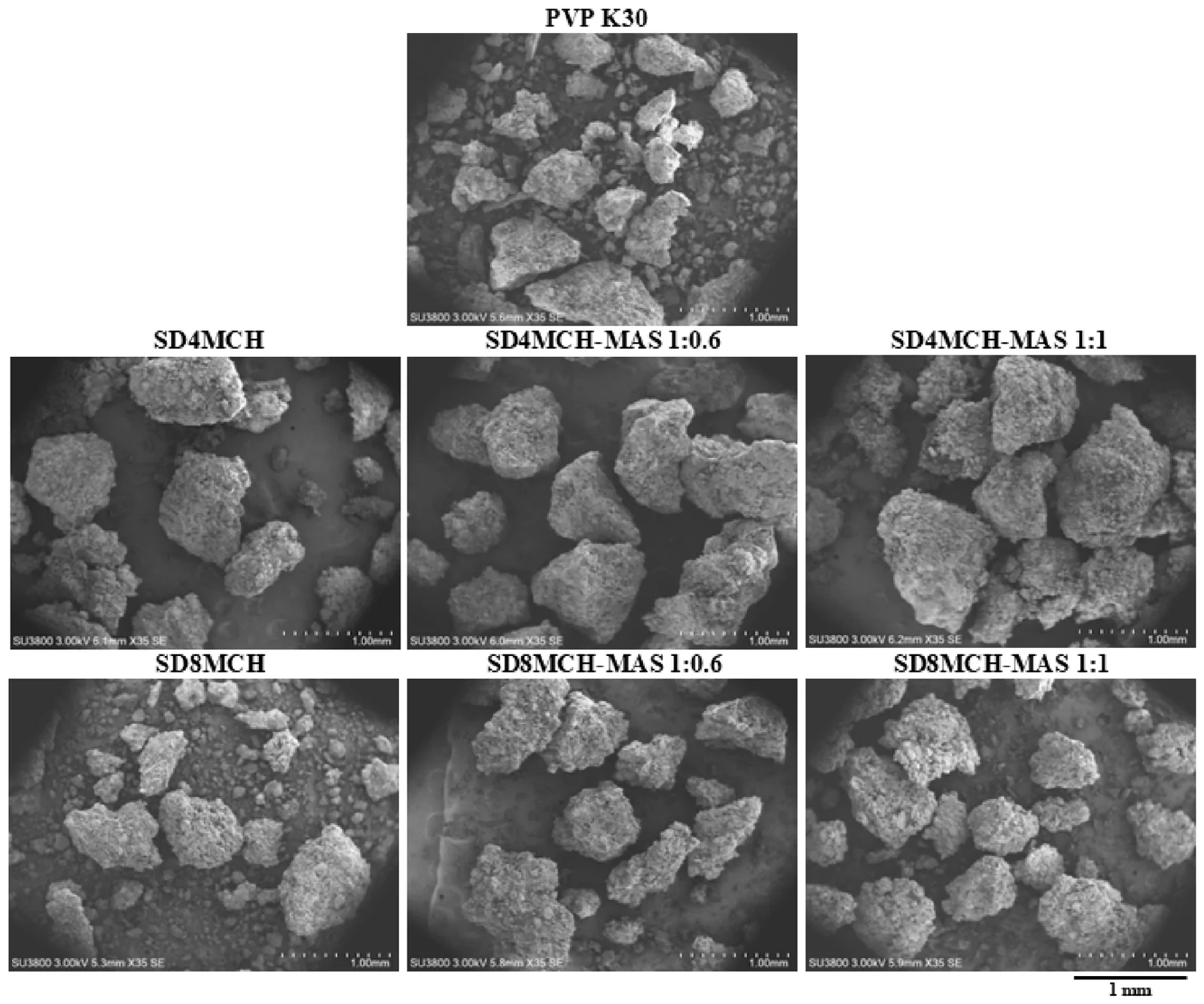
Particle morphology of lactose granules prepared using PVP K30, SDCH, and SDCH-MAS composites as a binder at a concentration of 2% *w*/*w*.

Figure 5 shows the particle morphologies of granules prepared with different binders. All granules exhibited an irregular shape with a rough surface, resulting from particle cracking during sieving. SEM images show that granules made with SD8MCH and SD8MCH-MAS composites had smaller particle sizes than those made with SD4MCH and SD4MCH-MAS composites. This result could be explained by the fact that high-MW CH showed greater granule strength than low-MW CH. Thus, higher force during secondary sieving may cause granule cracking, leading to smaller particles after sieving. Additionally, granules using PVP K30 were also smaller than those using SD4MCH, which could be explained by similar reasons.

The micromeritic characteristics of lactose granules prepared using a 2% *w*/*w* binder are reported in Table 3, where PVP K30, a pharmaceutical binder, was used for comparison in this study. The lactose granules presented similar values for bulk, tapped, and true densities. The Carr’s index of the granules was in the range of 5.33–10.75%, indicating an excellent level of flowability under static conditions [59], whereas the angle of repose, which was used for evaluating dynamic friction of powders [60], was 36.25–39.27°, suggesting a fair level of flowability. It could be observed that the MW of CH and CH-MAS ratios did not influence the densities and flowability of the lactose granules. Moreover, SDCH and SDCH-MAS composites provided similar characteristics of the lactose granules with PVP K30.

**Figure 6:**
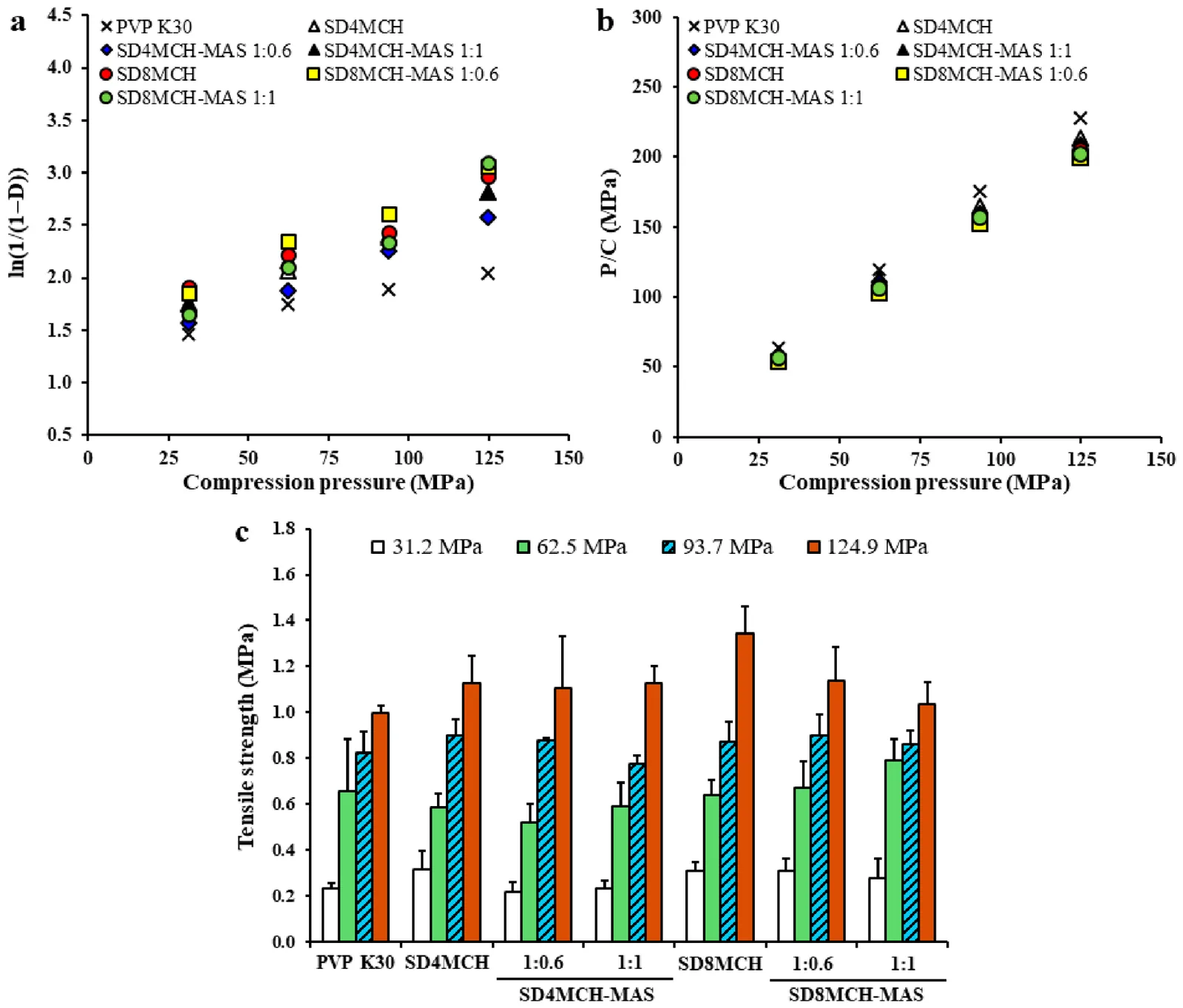
Heckel (**a**) and Kawakita (**b**) plots of lactose granules, and tensile strength (**c**) of lactose tablets prepared using various binders. Each point is the mean value of 3 tablets for Heckel and Kawakita plots, and the mean ± standard deviation of 3 tablets for tensile strength.

To evaluate the effect of the binder on the compressibility and plasticity of the lactose granules, tablets were prepared by compressing the granules at different compression pressures. The resulting out-die tablet thicknesses were measured, and the data were used to conduct Heckel plots, as shown in Figure 6a. The Heckel plot of the lactose granules with different binders showed good fitting, with R^2^ values greater than 0.96 in the range of 31.2–93.7 MPa compression pressures. The lactose granules prepared with PVP K30 yielded the lowest K value, 0.0067 1/MPa (Table 4). The K values of lactose monohydrate have been reported to be 0.0030 1/MPa, whereas that of pure PVP K30 was 0.0085 1/MPa [47]. This study suggested that using PVP K30 as a binder could increase the plasticity of lactose monohydrate under pressure. However, this granule showed the highest P_y_ value (yield pressure), indicating that plastic deformation began at a high compression pressure (149.25 MPa). The K values of the lactose granules prepared using SDCH and SDCH-MAS composites were in the range of 0.0083–0.0121 1/MPa, which were higher than those prepared using PVP K30 (Table 4). The granules using SD4MCH provided a greater K value than those using SD8MCH, indicating that the P_y_ value of SD4MCH (98.04 MPa) was lower than that of SD8MCH (120.48 MPa). This result suggested greater plasticity (lower yield pressure for deformation) in the lactose granules prepared using SD4MCH. Interestingly, the SDCH-MAS 1:0.6 ratio showed a higher K value than the SDCH and SDCH-MAS 1:1 ratios in both MWs of CH. This outcome resulted in a lower P_y_ value for the granules using an SDCH-MAS 1:0.6 ratio. However, increasing the MAS ratio decreased the P_y_ value compared with the SDCH-MAS 1:0.6 ratio, indicating that MAS increased granule plasticity. For the D_a_ parameter computed using Equation (4), it represented the total densification under low pressure, with the lactose granule value in the range 0.722–0.812. A similar D_a_ for the granules was obtained, suggesting that the effects of the MW of CH and MAS addition on the D_a_ parameter were not apparent in this study. This outcome may be due to the similar particle-size distribution of the granules prepared using the same method.

The Kawakita plots of the relationship between P/C and compression pressure are shown in Figure 6b. The parameters computed from this relationship, with R^2^ > 0.99, are listed in Table 4. The initial porosity (a) of the granules with SDCH and SDCH-MAS composites was similar, but greater than that with PVP K30. The P_k_ (yield strength) of the granules using PVP K30 was 7.699 MPa, whereas those using SD4MCH and SD8MCH were 7.402 and 7.474 MPa, respectively, indicating greater plasticity (lower resistance to volume change) in the granules with SDCH. The addition of MAS altered the P_k_ value. The granules with SDCH-MAS 1:0.6 composites showed lower P_k_ values than those with SDCH and SDCH-MAS 1:1 composites, regardless of the CH MW used, suggesting that SDCH-MAS 1:0.6 composites exhibit lower resistance to volume reduction under pressure. In the Heckel and Kawakita analyses, the granules with SDCH-MAS 1:0.6 composites showed lower yield pressure (P_y_) and yield strength (P_k_) than other granules, resulting in greater plasticity during deformation. Additionally, the hardness of the compressed tablets was measured and converted to tensile strength. The tensile strength of the tablets at different compression pressures is shown in Figure 6c. It can be seen that the tablets’ tensile strength increased with increasing compression pressure. However, this parameter of the tablets using different binders showed similar results and was not clear for comparison at the same pressure.

This study also found that particle strength (Figure 4) was related to the plastic deformation of the granules, using SDCH and SDCH-MAS composites as binders. The smaller the particle strength, the lower the yield pressure and yield strength for plastic deformation were obtained. Moreover, changes in yield pressure and yield strength with SD4MCH-MAS and SD8MCH-MAS composites were influenced by the granule particle strength.

### 3.3. Properties of PPN Granules and Tablets Using Various Binders

To investigate the binder performance of SDCH and SDCH-MAS composites in the presence of API, PPN was selected as a model drug. The properties of lactose granules containing PPN, prepared using different binders, are reported in Table 5. The bulk and tapped densities of the PPN granules were comparable with those of the lactose granules without PPN (Table 3). Moreover, the flowability of the PPN granules was at an excellent level using Carr’s index and a fair level using repose angle, which was similar to the lactose granules. These outcomes indicated that the incorporation of PPN did not influence the micromeritic properties of the lactose granules.

All granules could be compressed into a tablet at a compression pressure of 125 MPa. The thicknesses of the tablets ranged from 2.88 to 3.03 mm (Table 5). The PPN contents in the tablets were 97.2–106.2% *w*/*w*. For the hardness of the tablets, those using PVP K30 achieved a hardness of 5.9 kP. The SD4MCH and SD4MCH-MAS 1:0.6 composites exhibited comparable hardness and were similar to those using PVP K30. However, increasing the MAS ratio resulted in a significantly higher tablet hardness (*p* < 0.05) compared to using a binder with a smaller MAS ratio or without MAS. The improvement in tablet mechanical strength is likely due to MAS contributing to stronger interparticulate bonding during compression [27]. This phenomenon could be found in the use of SD8MCH and SD8MCH-MAS composites. The use of higher MW of CH tended to provide a lower tablet hardness, but there was no significant difference (*p* > 0.05) when compared with using lower MW of CH in this study. It could be explained that the greater particle strength of the granules when using high MW of CH (Figure 4) possibly presented a higher resistance to form a cold-welding process during deformation under compression pressure, which was similar to the results of the previous studies [15,61].

The disintegration time of the tablets in 0.1 N HCl is listed in Table 4. The PPN tablets, using PVP K30, had a disintegration time of 5.73 min. The mechanism of tablet disintegration involved a dissolution process due to the water-soluble properties of PPN, lactose, and PVP K30. The use of SD4MCH as a binder provided a statistically longer disintegration time (*p* < 0.05) than using PVP K30 due to the swelling property of CH in acidic medium, leading to gel formation and retardation of the disintegration process [62]. Using SD4MCH-MAS composites resulted in a shorter disintegration time compared to SD4MCH. Moreover, the shortest disintegration time was found when employing SD4MCH-MAS 1:1 composites, although this tablet presented the greatest hardness. This result was due to MAS having a swelling property, which has been applied as a disintegrant in tablets [63]. With SD8MCH, tablet disintegration time was significantly shorter (*p* < 0.05) than with SD4MCH, while tablet hardness did not differ significantly between the two (*p* > 0.05). This result suggested that CH swelling contributed to tablet disintegration. Huanbutta et al. [64] reported that matrix tablets prepared from CH with high MW swelled more than those of low MW. However, the binder concentration used in this study was 2% *w*/*w*, suggesting that hydrated SDCH could disperse in the mixture during granulation and distribute throughout the tablets after compression. When tablets were immersed in 0.1 N HCl, SDCH rapidly swelled and caused tablet disintegration. Thus, SD8MCH could create greater internal mechanical pressure in tablets than SD4MCH because of its greater swelling. This resulted in a shorter disintegration time of the tablets with SD8MCH. In addition, tablets containing SD8MCH-MAS 1:1 composites had a very short disintegration time, attributed to MAS’s disintegration properties.

**Figure 7:**
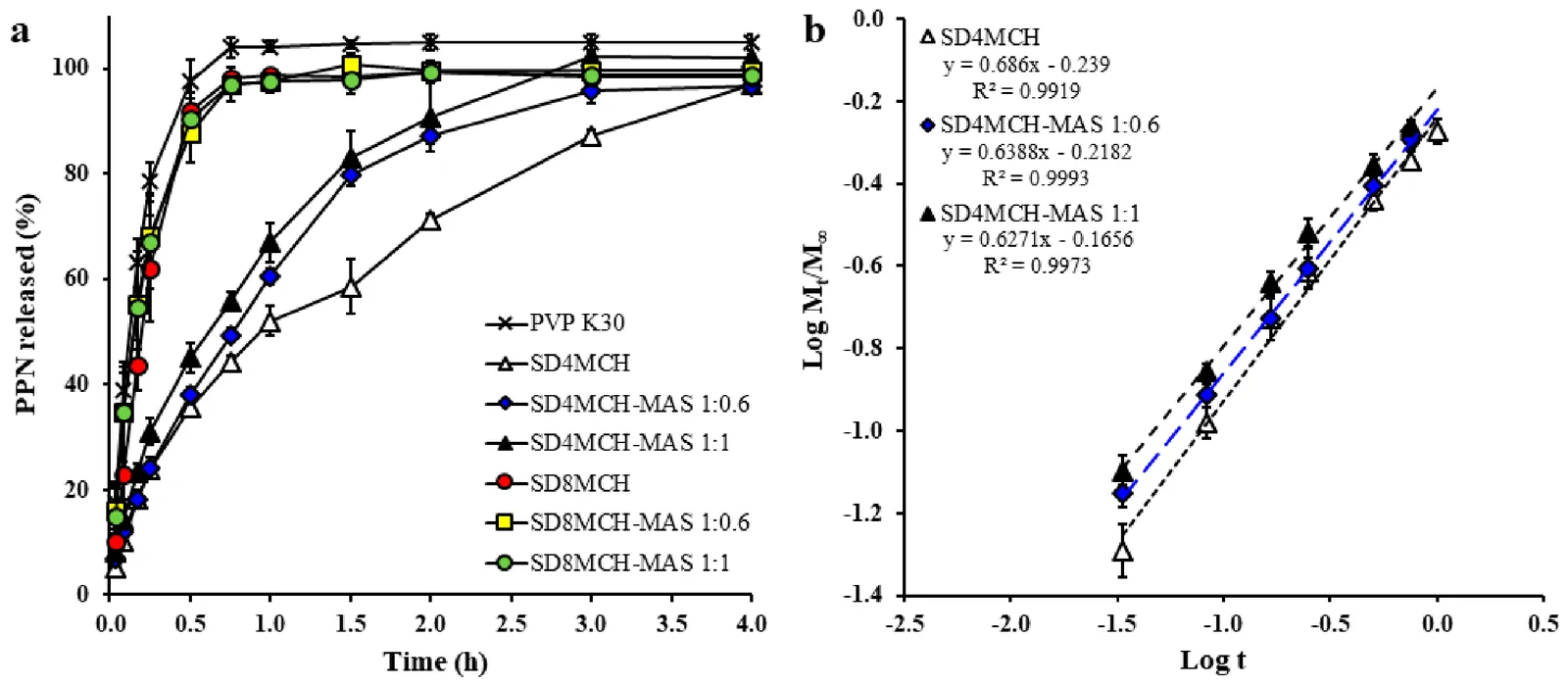
Drug release profiles (**a**) of the PPN tablets using lactose granules with different binders, and relationship between log M_t_/M_∞_ and log t (**b**) of the tablets prepared from the lactose granules with SD4CH and SD4MCH-MAS composites. The dissolution medium was 0.1 N HCl. Each point represents the mean ± standard deviation (*n* = 3).

**Figure 8:**
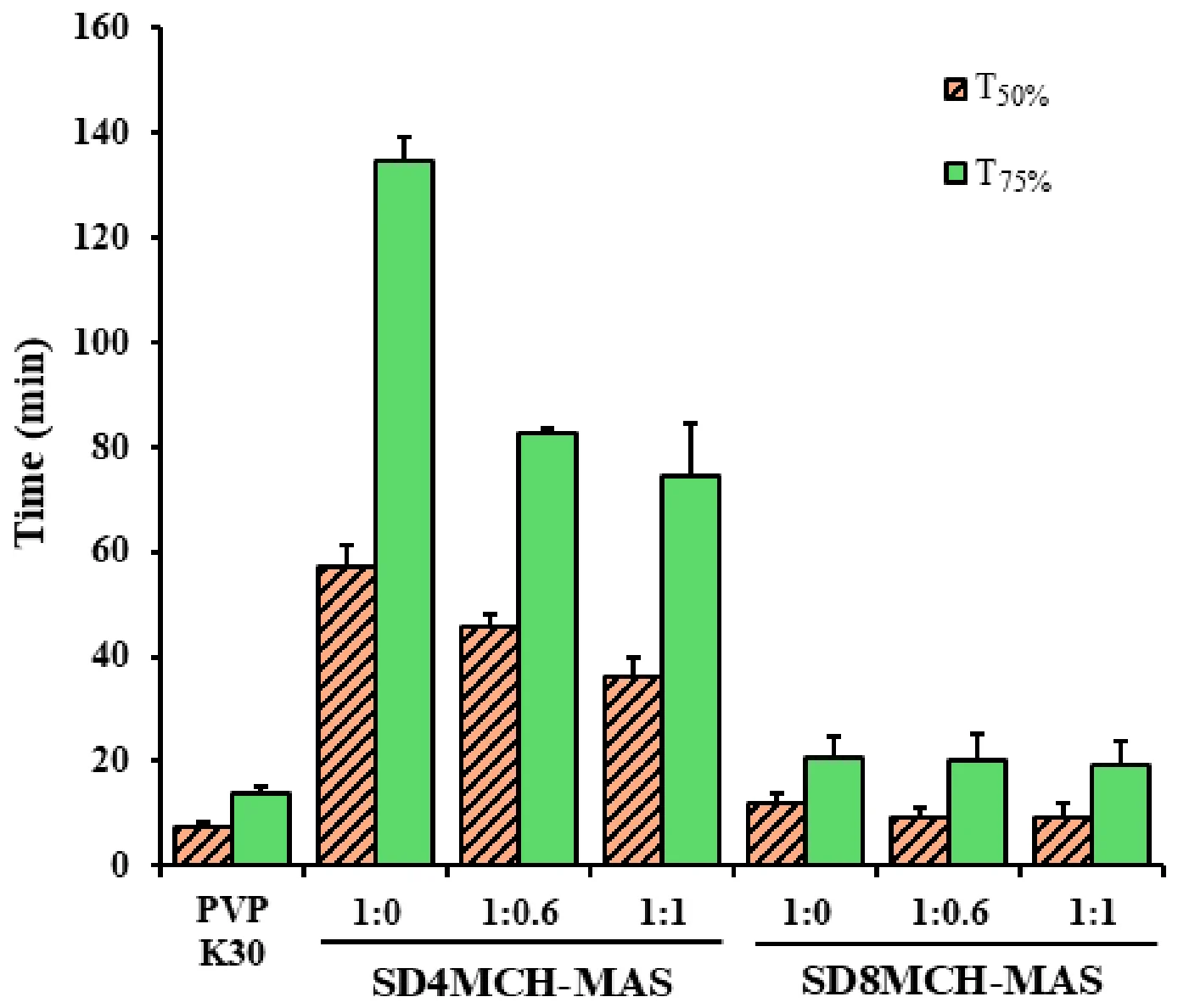
Times required to achieve 50% and 75% PPN release (T_50%_ and T_75%_) of tablets using lactose granules with different binders. Each value represents the mean ± standard deviation (*n* = 3).

The drug release profiles of the PPN tablets, using lactose granules with different binders, are presented in Figure 7a. The PPN tablets with PVP K30 exhibited a fast release pattern, with more than 80% of the drug released within 30 min. Using SD4MCH and SD4MCH-MAS composites resulted in a modified-release pattern of PPN, whereas a fast release of PPN was found when using higher MW of CH. The release parameters for comparison were T_50%_ and T_75%_, as presented in Figure 8. The longer the T_50%_ and T_75%_, the slower the PPN release was obtained. The SD4MCH exhibited significantly slower drug release (*p* < 0.05) compared to the SD4MCH-MAS composites, and an increase in the MAS ratio accelerated drug release. The tablets with SD8MCH and SD8MCH-MAS composites exhibited shorter T_50%_ and T_75%_ values than those with SD4MCH and SD4MCH-MAS composites, while achieving a comparable release of the drug.

The tablets containing SD4MCH and SD4MCH-MAS composites could form a swollen matrix to retard drug release because the agitation force in the dissolution test condition was obviously smaller than that of the disintegration test [65]. To explain the drug release mechanism of these tablets, the power law (Equation (7)) was applied. The relationship between log fractional release and log t is presented in Figure 7b. It can be observed that the relationship showed good linearity (R^2^ > 0.99) when the fractional release was not more than 0.6. The mean release exponent n, slope value, was 0.686, 0.639, and 0.627 (Figure 7b) for the PPN tablets using SD4MCH and SD4MCH-MAS composites at the ratios of 1:0.6 and 1:1, respectively. The results suggested that the tablets could swell in an acidic medium, and that PPN release involved anomalous transport mechanisms involving both drug diffusion and polymer swelling. The faster drug release in the presence of MAS occurred because MAS had a swelling property that could accelerate the swelling and erosion of the swollen tablets. In contrast, the tablets with SD8MCH showed immediate PPN release, suggesting that this tablet also disintegrated rapidly under the dissolution test conditions. Furthermore, the addition of MAS could also accelerate drug release. These findings suggest that using SDCH and SDCH-MAS composites as binders affected both tablet disintegration time and drug-release characteristics.

CH has been utilized as a binder in the wet granulation method for tablet preparation, requiring the dispersion of CH powder in an acidic solution. The resulting viscous CH dispersion was then mixed with a drug-excipient mixture to create a wet mass for granulation. The binding efficiency of CH was found to be superior to that of methyl cellulose and sodium carboxymethylcellulose [18]. Additionally, SDCH was developed for use as a binder. This SDCH was dispersed in water to form a viscous dispersion before being added to the drug-excipient mixture. Tablets formulated with SDCH exhibited slower drug release compared to those made with PVP, and the drug release rate decreased further as the amount of SDCH used increased [21]. This study introduces an alternative approach by using SDCH and SDCH-MAS composites in powder form for blending with PPN and lactose. Water was incorporated to induce the hydration and swelling of the SDCH and SDCH-MAS composites, which served as a binder in the formulation. The MWs of CH and the CH-MAS composites with different ratios in the SD powder form also influenced the characteristics of the PPN granules and tablets in this study.

### 3.4. Properties of ADP Crude Granules and Tablets Using Various Binders

According to the data in Table 5, using SD4MCH and SD4MCH-MAS 1:0.6 as a binder offered a greater hardness of the PPN tablets when compared to SD8MCH and SD8MCH-MAS 1:0.6. Thus, this part of the study investigated the application of SD4MCH and SD4MCH-MAS 1:0.6 composites as functional binders in ADP crude tablets, focusing on their ability to support tablet formation with minimal lactose as a diluent. The yield of ADP granules obtained from all formulations was 84–93% *w*/*w*. As shown in Table 6, the ADP:lactose granules at ratios of 100:0 and 75:25, using SD4MCH as a binder, exhibited low bulk and tapped densities. In contrast, increasing the lactose amount to the 50:50 ADP:lactose ratio increased both densities. All ADP:lactose granules presented similar Carr’s index and repose angle values. Moreover, a similar moisture content was obtained. Apart from granules, the ADP:lactose 100:0 tablets exhibited the lowest hardness, resulting in a high value of friability and a very short disintegration time in 0.1 N HCl. The ADP:lactose 75:25 tablets showed a lower %RSD in weight, greater hardness, and lower friability than the 50:50 ADP:lactose tablets. Thus, a 75:25 ADP:lactose mixture was used to test PVP K30, SD4MCH, and SD4MCH-MAS 1:0.6 composites as binders.

**Figure 9:**
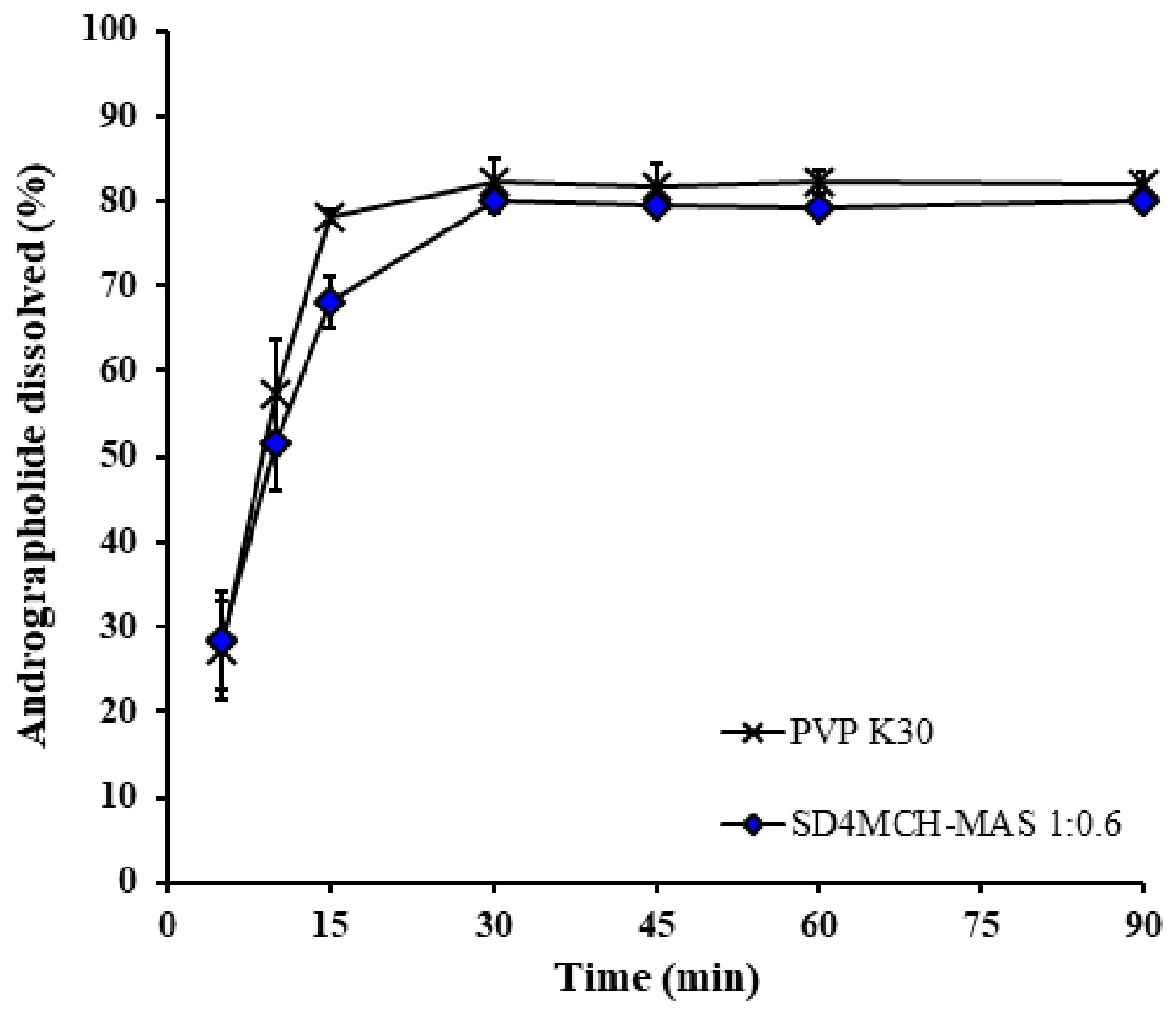
Andrographolide dissolution profiles of ADP crude tablets (75:25 ADP:lactose ratio) using PVP K30 and SD4MCH-MAS composites as a binder. Each point represents the mean ± standard deviation (*n* = 3).

The ADP:lactose 75:25 granules prepared with PVP K30 and SD4MCH-MAS composites showed higher bulk and tapped densities than those prepared with SD4MCH (Table 6). SD4MCH-MAS composites showed lower Carr’s index and repose angle values; however, PVP K30 achieved lower moisture content. For the tablet features, tablets using SD4MCH-MAS composites exhibit significantly greater hardness (*p* < 0.05) than those using PVP K30 and SD4MCH. Tablets using SD4MCH showed the lowest hardness. Additionally, tablets using PVP K30 showed shorter disintegration time than those using SD4MCH and SD4MCH-MAS composites. Furthermore, the tablets prepared using SD4MCH were the only formulation to show a friability value higher than 1%. A general criterion for tablet friability is a weight loss of less than 1.0%, as required in the US Pharmacopeia [66]. During mechanical rotation, tablets with SD4MCH showed chipping of the tablet surface and edges. In contrast, tablets with PVP K30 and SD4MCH-MAS composites showed less chipping because they were harder than SD4MCH tablets. Based on these results, the tablets using PVP K30 and SD4MCH-MAS composites were applied to test andrographolide dissolution, and the dissolution profile is shown in Figure 9. Both tablets showed rapid andrographolide dissolution. The tablets using PVP K30 gave significantly higher andrographolide dissolution (*p* < 0.05) than those using SD4MCH-MAS composites at 15 min of the test. However, both tablets reached a plateau at 30 min of the test, at which the amount of andrographolide was not statistically different (*p* > 0.05).

The tablets prepared using PVP K30 and SD4MCH-MAS composites contained ADP crude powder at 364 mg/tablet (theoretical calculation), which included 6.66 mg of andrographolide. This amount is based on the andrographolide content in the ADP crude powder, which was determined to be 1.83% *w*/*w* by HPLC. According to the dissolution study results, the amount of andrographolide that dissolved from the tablets using PVP K30 and SD4MCH-MAS composites was 5.45 ± 0.18 mg and 5.33 ± 0.08 mg at 30 min of the test, corresponding to andrographolide dissolved of 81.80 ± 2.71% and 79.96 ± 1.27%, respectively. The dissolution testing in this study demonstrated that the tablets obtained could disintegrate rapidly, leading to the rapid dissolution of andrographolide from the ADP crude powder. Additionally, literature reviews of dissolution tests on marketed ADP crude capsules (seven products) in an acidic medium reported that the amount of andrographolide dissolved ranged from 3.19 mg to 11.19 mg per capsule over 100 min [67]. Another study found that marketed ADP crude capsules from three products had an andrographolide dissolution rate exceeding 80% within 45 min in 0.1 N HCl [68].

Although the Thai Herbal Pharmacopeia does not specify a standard for andrographolide dissolution, the ADP crude tablets developed in this study demonstrated an acceptable dissolution profile, with more than 75% of the andrographolide dissolving in 0.1 N HCl within 30 min. Therefore, the use of SD4MCH-MAS composites at a ratio of 1:0.6 as a binder proved effective for producing acceptable ADP crude tablets in this study, and the tablets offered similar characteristics to those produced with PVP K30.

### 3.5. Considerations for Scale-Up Production

Due to the laboratory scale of this study, establishing a definitive scale-up process for tablet production using lactose as a diluent and SDCH-MAS nanocomposites as a binder remains challenging. SDCH-MAS nanocomposites can be produced on a larger scale via spray drying, as outlined in Section 2.2. Tablet formulations, such as an ADP:lactose ratio of 75:25 with 2% *w*/*w* SDCH-MAS nanocomposites, can be blended using a high shear mixer [69]. A binding fluid (water) should be sprayed onto the mixture to achieve uniform wetting until a wet mass is formed. The volume of water or the binding fluid:solid ratio is a critical parameter [70]. The wet mass can be sieved to form granules using an oscillating granulator, followed by drying in a hot air oven. Dried granules may be further reduced in particle size using an appropriate sieve and the oscillating granulator. Key properties, including particle size distribution, density, and flowability of the granules, should be evaluated [69,70]. If necessary, extragranular disintegrant may be added and blended with the granules using a Y-shaped mixer. The resulting mixtures can then be blended with a lubricant (1% *w*/*w* magnesium stearate) and compressed using a rotary tableting machine. Quality control assessments should include weight variation, content uniformity, friability, hardness, disintegration time, and drug dissolution [69].

## 4. Conclusions

Nanocomposites of CH and MAS, prepared by spray drying, could rapidly swell in water and form a viscous gel. The SDCH-MAS gels gave lower viscosity than the SDCH gels, and the flow type and viscosity of the gels depended on the MW of CH used. Increasing the MAS ratio reduced the gel’s viscosity. Both the SDCH and SDCH-MAS composites demonstrated binding properties that effectively produced lactose granules. The density and flowability of these lactose granules were comparable to those of granules produced with PVP K30. Using SDCH and SDCH-MAS composites could reduce yield pressure and yield strength during plastic deformation compared to PVP K30. The SDCH-MAS 1:0.6 composites exhibited the lowest yield pressure and yield strength during compression. Moreover, the particle strength of the granules also affected the plastic deformation when these composites were used.

The lactose granules containing PPN, using SDCH and SDCH-MAS composites as binders, exhibited good flowability and could be compressed into tablets. The PPN tablets present acceptable physical properties. Using SD4MCH-MAS 1:1 and SD8MCH-MAS 1:1 composites caused the highest tablet hardness. Increasing MAS ratios could reduce the disintegration time of tablets in an acidic medium. Tablets with a lower MW of CH showed longer disintegration times, leading to modified PPN release in an acidic medium, controlled by drug diffusion and polymer swelling. MAS could accelerate drug release. In contrast, the tablets containing SD8MCH and SD8MCH-MAS composites showed rapid release of PPN.

The ADP crude tablets were formulated with an ADP:lactose ratio of 75:25, resulting in a high crude powder content. The SD4MCH-MAS composites exhibited good micromeritic properties, similar to those of granules using PVP K30. Tablets created with both binders achieved acceptable hardness and friability, with more than 75% andrographolide dissolution in an acidic medium within 30 min. These findings suggest that SDCH-MAS composite gels can serve as effective binders for lactose granules prepared by wet granulation and demonstrate potential use with other poorly compressible drugs and herbal crude powders for tablet production. However, further studies on scale-up production and stability of these granules and tablets are required.

## Figures and Tables

**Table 1:** Components of ADP crude granules and volume of purified water used for preparation.

Components	ADP:Lactose				
	100:0	75:25	50:50	75:25	75:25
	SD4MCH	SD4MCH	SD4MCH	PVP K30	SD4MCH-MAS 1:0.6
ADP crude powder (g)	98.0	73.5	49.0	73.5	73.5
Lactose monohydrate (g)	-	24.5	49.0	24.5	24.5
Binder (g)	2.0	2	2	2	2
Purified water used (mL)	113.0	98.4	76.0	87.0	97.0

**Table 2:** Rheological parameters and pH of SDCH and SDCH-MAS gels.

Gels	pH	N	Viscosity Coefficient ((dyne cm^−2^)^N^ s)	Viscosity at 20.4 s^−1^ Shear Rate (mPa s)
SD4MCS-MAS				
1:0	5.91 ± 0.02	1.104 ± 0.011	2.60 ± 0.10	178.50 ± 2.27
1:0.6	5.62 ± 0.01	1.031 ± 0.162	0.69 ± 0.03	62.50 ± 2.01
1:1	5.93 ± 0.01	1.076 ± 0.036	0.58 ± 0.05	48.66 ± 0.78
SD8MCS-MAS				
1:0	5.83 ± 0.01	1.218 ± 0.011	14.01 ± 0.88	488.56 ± 2.83
1:0.6	5.91 ± 0.01	1.325 ± 0.007	15.84 ± 0.57	388.66 ± 2.24
1:1	5.95 ± 0.01	1.308 ± 0.056	4.33 ± 0.88	149.17 ± 5.76

Data are the mean ± standard deviation, *n* = 3.

**Table 3:** Micromeritic properties of lactose granules prepared using different binders.

Binder	Bulk Density (g/ml)	Tapped Density (g/ml)	True Density (g/ml)	Carr’s Index (%)	Angle of Repose (°)
PVP K30	0.563 ± 0.016	0.596 ± 0.014	1.470 ± 0.011	5.61 ± 1.76	37.84 ± 0.36
SD4MCH–MAS					
1:0	0.552 ± 0.006	0.592 ± 0.007	1.412 ± 0.006	6.75 ± 1.03	36.25 ± 0.96
1:0.6	0.536 ± 0.016	0.566 ± 0.011	1.448 ± 0.005	5.33 ± 1.63	36.83 ± 0.20
1:1	0.526 ± 0.009	0.584 ± 0.007	1.401 ± 0.011	9.93 ± 0.87	38.52 ± 0.50
SD8MCH–MAS					
1:0	0.514 ± 0.005	0.573 ± 0.006	1.389 ± 0.009	10.29 ± 0.10	37.73 ± 0.48
1:0.6	0.500 ± 0.008	0.549 ± 0.011	1.400 ± 0.006	8.89 ± 0.97	38.76 ± 0.49
1:1	0.509 ± 0.009	0.570 ± 0.017	1.400 ± 0.002	10.75 ± 1.15	39.37 ± 0.43

Data are the mean ± standard deviation, *n* = 3.

**Table 4:** Heckel and Kawakita parameters of lactose granules prepared by using different binders.

Lactose Granules	Heckel Parameters				Kawakita Parameters		
	K (1/MPa)	A	D_a_	P_y_ (MPa)	a	b	P_k_ (MPa)
PVP K30	0.0067 (R^2^ = 0.9611)	1.2784	0.722	149.25	0.5851 (R^2^ = 0.9998)	0.1304	7.669
SD4MCH-MAS							
1:0	0.0102 (R^2^ = 1.0000)	1.4298	0.761	98.04	0.6215 (R^2^ = 0.9998)	0.1351	7.402
1:0.6	0.0111 (R^2^ = 0.9966)	1.2071	0.701	90.09	0.6402 (R^2^ = 0.9999)	0.1397	7.158
1:1	0.0103 (R^2^ = 0.9807)	1.4661	0.769	97.09	0.6403 (R^2^ = 0.9997)	0.1385	7.220
SD8MCH-MAS							
1:0	0.0083 (R^2^ = 0.9889)	1.6705	0.812	120.48	0.6534 (R^2^ = 0.9996)	0.1338	7.474
1:0.6	0.0121 (R^2^ = 0.9678)	1.5172	0.781	82.64	0.6650 (R^2^ = 0.9997)	0.1426	7.013
1:1	0.0110 (R^2^ = 0.9682)	1.3388	0.738	90.91	0.6609 (R^2^ = 0.9997)	0.1329	7.526

**Table 5:** Characteristics of lactose granules containing PPN and PPN tablets prepared using different binders.

Binder	Granule Properties ^a^				Tablet Properties			
	Bulk Density (g/mL)	Tapped Density (g/mL)	Carr’s Index (%)	Repose Angle (°)	Thickness ^b^ (mm)	Hardness ^b^ (kP)	Drug Content ^a^ (%*w*/*w*)	Disintegration Time ^a^ (min)
PVP K30	0.501 ± 0.014	0.543 ± 0.020	7.80 ± 0.87	37.84 ± 0.40	3.03 ± 0.03	5.90 ± 0.31	97.15 ± 1.48	5.73 ± 0.54
SD4MCH–MAS								
1:0	0.519 ± 0.003	0.559 ± 0.004	7.15 ± 0.54	38.97 ± 0.45	3.02 ± 0.02	5.06 ± 0.40	103.33 ± 1.15	20.54 ± 0.58
1:0.6	0.546 ± 0.010	0.608 ± 0.014	10.28 ± 2.69	38.97 ± 0.51	3.03 ± 0.01	5.22 ± 0.40	106.20 ± 1.74	19.64 ± 0.25
1:1	0.536 ± 0.009	0.606 ± 0.002	11.44 ± 1.60	39.10 ± 0.10	2.96 ± 0.01	7.96 ± 0.49	102.83 ± 2.41	18.02 ± 1.60
SD8MCH–MAS								
1:0	0.529 ± 0.005	0.592 ± 0.013	10.59 ± 1.76	39.45 ± 0.50	3.01 ± 0.01	4.46 ± 0.23	104.08± 0.61	7.81 ± 0.23
1:0.6	0.533 ± 0.011	0.586 ± 0.008	8.94 ± 0.51	39.88 ± 0.34	2.99 ± 0.01	4.24 ± 0.23	103.34 ± 0.43	7.19 ± 0.23
1:1	0.530 ± 0.003	0.601 ± 0.002	11.81 ± 0.72	40.03 ± 0.91	2.88 ± 0.01	7.08 ± 0.39	102.61 ± 1.88	2.33 ± 0.08

^a^ Data are the mean ± standard deviation, *n* = 3; ^b^ Data are the mean ± standard deviation, *n* = 5.

**Table 6:** Characteristics of ADP crude granules and tablets using various binders.

Characteristics	ADP:Lactose				
	100:0	75:25	50:50	75:25	75:25
	SD4MCH	SD4MCH	SD4MCH	PVP K30	SD4MCH-MAS 1:0.6
Granules					
Bulk density ^a^ (g/ml)	0.302 ± 0.028	0.302 ± 0.003	0.353 ± 0.004	0.330 ± 0.006	0.335 ± 0.003
Tapped density ^a^ (g/ml)	0.332 ± 0.033	0.330 ± 0.006	0.382 ± 0.004	0.366 ± 0.008	0.366 ± 0.004
Carr’s index ^a^ (%)	9.05 ± 0.83	8.55 ± 0.94	7.64 ± 0.98	9.89 ± 0.19	8.38 ± 0.08
Angle of repose ^a^ (°)	36.55 ± 1.01	35.46 ± 0.91	35.11 ± 0.97	36.87 ± 0.21	33.91 ± 0.17
Moisture content ^a^ (%)	6.01 ± 0.14	5.50 ± 0.25	4.00 ± 0.17	4.99 ± 0.05	7.15 ± 0.23
Tablets					
Weight ^b^ (mg)	433.78 ± 4.0 (%RSD = 0.92)	485.42 ± 2.02 (%RSD = 0.42)	511.15 ± 9.09 (%RSD = 1.78)	488.34 ± 7.66 (%RSD = 1.57)	506.14 ± 8.15 (%RSD = 1.61)
Thickness ^c^ (mm)	3.36 ± 0.01	3.37 ± 0.03	3.27 ± 0.02	3.27 ± 0.01	3.30 ± 0.02
Hardness ^c^ (kP)	1.67 ± 0.13	4.23 ± 0.21	3.64 ± 0.64	4.60 ± 0.55	5.79 ± 0.57
Friability (%)	10.75	1.08	1.40	0.81	0.82
Disintegration time ^a^ (min)	0.32 ± 0.02	8.30 ± 0.70	11.44 ± 0.91	7.41 ± 0.14	8.27 ± 0.72

^a^ Data are the mean ± standard deviation, *n* = 3. ^b^ Data are the mean ± standard deviation, *n* = 20. ^c^ Data are the mean ± standard deviation, *n* = 5.

## Data Availability

The original contributions presented in this study are included in the article. Further inquiries can be directed to the corresponding author.
